# Au nanoparticles modified CuO nanowire electrode based non-enzymatic glucose detection with improved linearity

**DOI:** 10.1038/s41598-020-67986-4

**Published:** 2020-07-10

**Authors:** Ashwini Kumar Mishra, Deepak Kumar Jarwal, Bratindranath Mukherjee, Amit Kumar, Smrity Ratan, Manas Ranjan Tripathy, Satyabrata Jit

**Affiliations:** 1 Department of Electronics Engineering, Indian Institute of Technology (BHU) Varanasi, Varanasi, 221005 India; 2 Department of Metallurgical Engineering, Indian Institute of Technology (BHU) Varanasi, Varanasi, India

**Keywords:** Biochemistry, Biological techniques, Biotechnology

## Abstract

This paper explores gold nanoparticle (GNP) modified copper oxide nanowires(CuO NWs)based electrode grown on copper foil for non-enzymatic glucose detection in a wide linear ranging up to 31.06 mM, and 44.36 mM at 0.5 M NaOH and 1 M NaOH concentrations. The proposed electrode can be used to detect a very low glucose concentration of 0.3 µM with a high linearity range of 44.36mM and sensitivity of 1591.44 µA mM^−1^ cm^−2^. The electrode is fabricated by first synthesizing Cu (OH)_2_ NWs on a copper foil by chemical etching method and then heat treatment is performed to convert Cu (OH)_2_ NWs into CuO NWs. The GNPs are deposited on CuO NWs to enhance the effective surface-to-volume ratio of the electrode with improved catalytic activity. The surface morphology has been investigated by XRD, XPS, FE-SEM and HR-TEM analysis. The proposed sensor is expected to detect low-level of glucose in urine, and saliva. At the same time, it can also be used to measure extremely high sugar levels (i.e. hyperglycemia) of ~ 806.5454 mg/dl. The proposed sensor is also capable of detecting glucose after multiple bending of the GNP modified CuO NWs electrode. The proposed device is also used to detect the blood sugar level in human being and it is found that this sensor’s result is highly accurate and reliable.

According to the world health organization (WHO), the number of diabetic patients is expected to reach up to 500 million by 2030^1,2^ in the world. Diabetic causes some severe complications like heart attack, stroke, kidney failure, vision loss and nerve damage^3^. The normal range of blood glucose concentration lies in the range of 80–120 mg/dl (4.4–6.6 mM) for a healthy person^3^. In general, blood glucose levels greater than 126 mg/dl when fasting and greater than 200 mg/dl at 2 h after meals are considered to be hyperglycemia^3^ which is dangerous from the health point of view. On the other hand, glucose level below 54 mg/dL is known as hypoglycemia which also needs immediate medical attention. Thus, it is required, especially for the diabetic persons with glucose levels above the normal range, to check the glucose level in blood regularly. As a result, glucose sensors have covered nearly 85% of the entire biosensors markets^3^. However, accurate and fast assessment of glucose level in blood is still challenge in biological and medical analysis^4,5^. Different techniques have been explored in electronic biosensors for detecting various chemical analytes like glucose, cholesterol, other fluids, gases etc^6–11^. In general, the resonance and capillary-zone electrophoresis methods are used in electronic glucose sensors^11–13^. However, the electrochemical based glucose level estimation techniques have drawn considerable attention of the researchers due to its natural simplicity, high sensitivity and good selectivity^14–16^.

Electrochemical glucose sensors are of two types depending on the electro-catalyst used on the sensor electrodes^10^. The first is enzymatic type glucose sensors that requires an enzyme like glucose oxidase (GOx), GDH, etc. on the electrode while the second type is called non-enzymatic glucose sensors which requires no enzymes in electrode for detection^10,17,18^. Although the enzymatic glucose sensors possess high sensitivity, high selectivity, and low detection limit^19^, but they suffer from poor stability resulted from variations of operating temperatures, pH values and relative humidity. Further, the enzymatic glucose sensors are costlier than the non-enzymatic glucose sensors due to requirement of costly enzymes ^20–23^. Thus, a significant emphasis has been given on the development of low-cost, accurate and fast-response based non-enzymatic glucose sensors^24–26^.

It is reported that performance improvement of such non-enzymatic glucose sensors can be achieved using electrodes with high conductivity, large specific surface area, high stability, good selectivity and good reproducibility with high capability of effective electron transfer from the electro-catalyst to the conductive surface of the electrode^27^. Several noble metals such as Pd, Au and Pt as well as their composites like Pt–Pd, Pt-Au, Au–Pd etc. have explored for glucose sensing^28–30^. However, the cost of these metals have encouraged to explore the possibility of metal oxides such as CuO, NiO, ZnO, Cu_2_O, MnO_2_, Fe_2_O_3_, SnO_2_ and Ag_2_O for developing low-cost non-enzymatic glucose^31^. Among them, CuO has been widely used for developing low-cost glucose sensors due to its good electrochemical and electro-catalytic properties, and abundant availability^11,16,25,27,32,33^. The intrinsically p-type semiconductor CuO with a band-gap of ~ 1.2 eV has also been extensively used in electrochemical sensors, photoelectric devices, gas sensors and lithium-ion batteries due to its interesting electrical and optical properties^15,34–36^. Various CuO nanostructures (e.g. nanowires, nanorods, nano flower, etc.) grown on a copper foil using easy and simple synthesis techniques have been reported for low-cost glucose sensors with a fast response, high sensitivity, and stable detection due to their enhanced catalytic property over the other metal oxide nanostructures^11^.

Li et al.^27^ have synthesized CuO nanowires (NWs) on 3-D copper foam by anodization process for glucose detection. They^27^ have achieved high sensitivity and wider linear range up to 18.8 mM at a concentration of 1 M NaOH. The high catalytic activity of the metal oxide and high conductivity of the noble metals have also encouraged to use nanocomposites of ≥ 95% metal oxide and ≤ 5% noble metal for glucose-sensing applications. Li et al. ^37^ have tried Au/CuO (metal and metal oxide)nano-composite (nano-cauliflower)to sense glucose for the first time.

They^37^ have achieved a detection limit of 0.3 µM, the sensitivity of 708.7 µA mM^−1^ cm^−2^ and linear range from 0.0001 mM to 30 mM at a concentration of 1 M of NaOH. Xio et al.^38^ have used Au/CuO Nanohybrids to get a sensitivity of 374 µA mM^−1^ cm^−2^ with linearity up to 12 mM at a concentration of 0.1 M NaOH (pH = 13) while Wang et al^19^ have got the sensitivity of 709.52µA mM^−1^ cm^−2^ and linearity up to 8 mM at a concentration of 0.1 M NaOH. The nano-composite of CuO (metal oxide) nanostructure and Au (metal) nanostructure shows the enhancement of conductivity to accelerate the rate of electron transfer, selectivity and sensitivity of glucose sensing^37^. In such glucose sensors, the direct electron transfer property of CuO NWs acts as catalysts whereas the large surface-to-volume ratio of Au nanoparticles can act as co-catalyst for enhancing the linearity and sensitivity in a drastic manner of the glucose sensors.

There are still challenges to develop glucose sensors with a wide range of linearity and high sensitivity for detecting the glucose levels in moderate to severe diabetic patients. Recently, Mishra et al.^39^ have observed a significant increase in sensitivity, linearity, and selectivity in gold nanoparticles (GNPs) modified CuO NWs based glucose sensors at low-concentration of 0.1 M of NaOH solvent. In this article, we have demonstrated the improvement of linearity and sensitivity of the GNPs modified CuO NWs electrode based glucose sensor using the higher levels of electrolyte (NaOH) concentrations at 0.5 M and 1 M.

## Results and discussions

### Characterizations of electrodes

**Figure 1:**
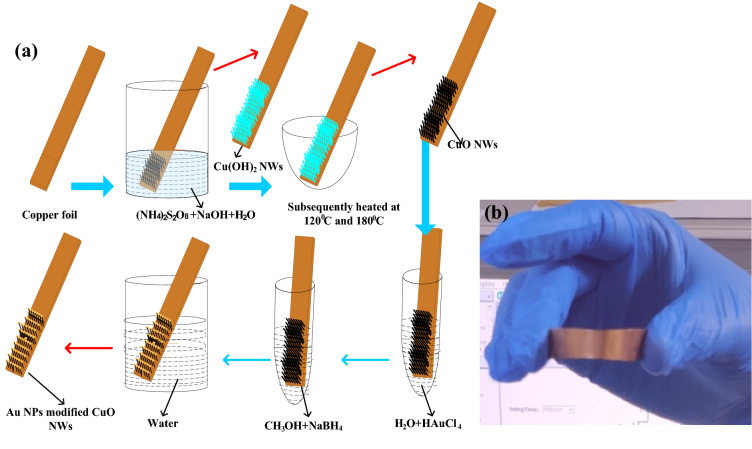
(**a**) Fabrication step of gold modified CuO NWs on Cu foil electrode, and (**b**) Bendable copper foil.

**Figure 2:**
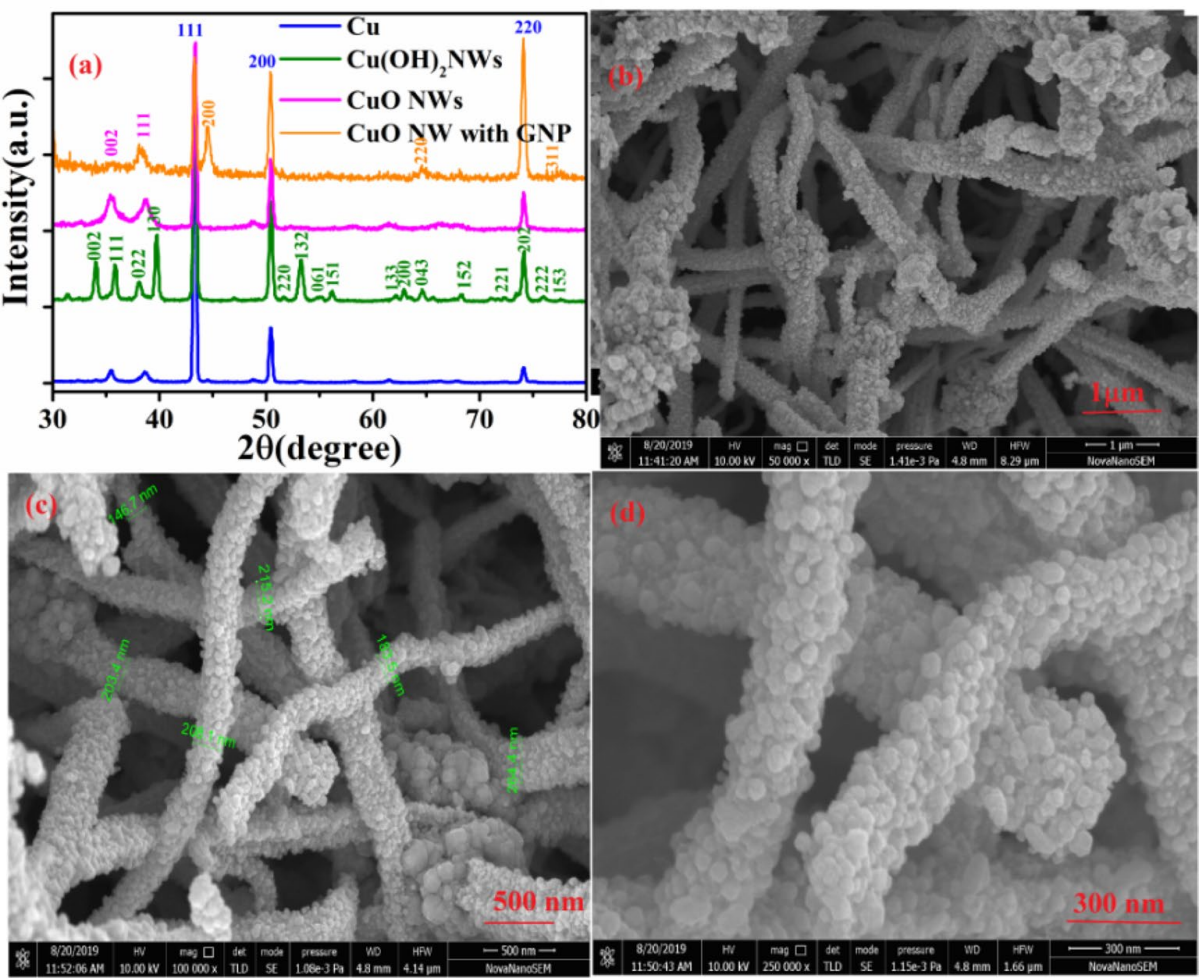
(**a**) XRD pattern of as synthesis Cu foil electrode, Cu(OH)_2_NWs, CuONWs and CuO NWs with GNP. (**b**) Low resolution of FESEM image of CuO NWs with GNP. (**c**) Moderate-resolution of FESEM image of CuO NWs with GNP. (**d**) High resolution of FESEM image of CuO NWs with GNP.

Synthesis and electorde fabrication is briefly illustrated in Fig. 1(a) along with detail explanation in experimental sections. Figure 2a shows the XRD patterns of Cu foil, Cu(OH)_2_ NWs, CuO NWs, and CuO NWs with GNP which are compared with standard JCPDS file numbers 04–0836, 35–0505, 80–1917, and 04–0784 of Cu, Cu(OH)_2_, CuO NWs, and GNPs modified CuO NWs, respectively. The crystalline sharp peaks found at 43.37 degrees, 50.61 degrees, and 74.27 degrees correspond to (111), (200) and (220) of FCC Cu confirming composition of the foil. The other two minor peaks at 35.39 degrees and 38.65 degrees are due to impurities of native oxide of copper. The XRD of Cu(OH)_2_ NWs sample also shown in Fig. 2(a) matches well with the orthorhombic phase (JCPDS 35–0505) thereby confirming the successful growth of Cu(OH)_2_ NWs on the Cu foil. Heating induced de hydroxylation leadsto re-crystallization and formation of CuO nanowires as evident from the XRD plot (JCPDS 80–1917) which is also shown in Fig. 2a. The comparison of the XRD pattern of the CuO NWs with GNP in Fig. 2a with those of CuO NWs confirm that other minor peaks in the XRD of the CuO NWs with GNP have resulted from the small amount of gold nanoparticles (GNP) uniformly grown on the CuO NWs.The FE-SEM image obtained for low magnification to high magnification for CuO NWs with GNP grown on Cu foil is shown in Fig. 2b–d.

The Field Emission Scanning electron microscopy (FE-SEM) images to illustrate morphology of CuO NWs with GNP under study are shown in Fig. 1b with low resolution. Moderate and high resolutions of CuO NWs with gold nanoparticles are shown in Fig. 2c–d. On the basis of Fig. 2c–d the diameter of the nanowire was observed in the range of 150–250 nm.

HRTEM image analysis gives us better investigations of the surface morphology of CuO NWs with GNP as shown in Supplementary Fig. S1. The free-standing of TEM images of CuO NWs with GNP is obtained after scrapping from the Cu substrate shown in Supplementary Fig. S1(a). The image of Supplementary Fig. S1(b) clearly shows the dark spot of well-distributed gold nano-particles on CuO NWs. In Supplementary Fig. S1(b) the presence of dark spot was due to higher atomic weight of gold (Au) in comparisons Cu and O. The average diameter of gold nanoparticles are found in the range of 10–30 nm and the diameter of the CuO NWs are found in the range of 100–150 nm. The selected area electron diffraction (SAED) pattern of the gold CuO NWs with GNP from the scanning image area is shown in Supplementary Fig. S1(c). The diameter of the first three circles corresponds to CuO (202), CuO (111)/Au (111), and CuO (002), with a d-spacing of 1.58 Å, 2.32 Å, and 2.5 Å, respectively. Further, the image is magnified as shown in Supplementary Fig. S1(d) and S1 (e) where dark spot shows the gold (Au) Nanoparticles and the white spot shows the presence of CuO NWs. Supplementary Fig. S1(f) shows the lattice spacing measurements for dark and white regions as 2.32 Å, and 2.5 Å, respectively which confirm the presence of Au (111) and CuO (002). Bright field TEM image of CuO NWs has been shown in Supplementary Fig. S2. There are no dark spot in the Supplementary Fig. S2(a) to (c), as it was present in Supplementary Fig. S1. SAED pattern shown in Supplementary Fig. S2(d) also confirms the absences of gold in the CuO NWs. Material composition and EDS spectra of the CuO NWs with GNP are shown in Supplementary Fig. S4.

**Figure 3:**
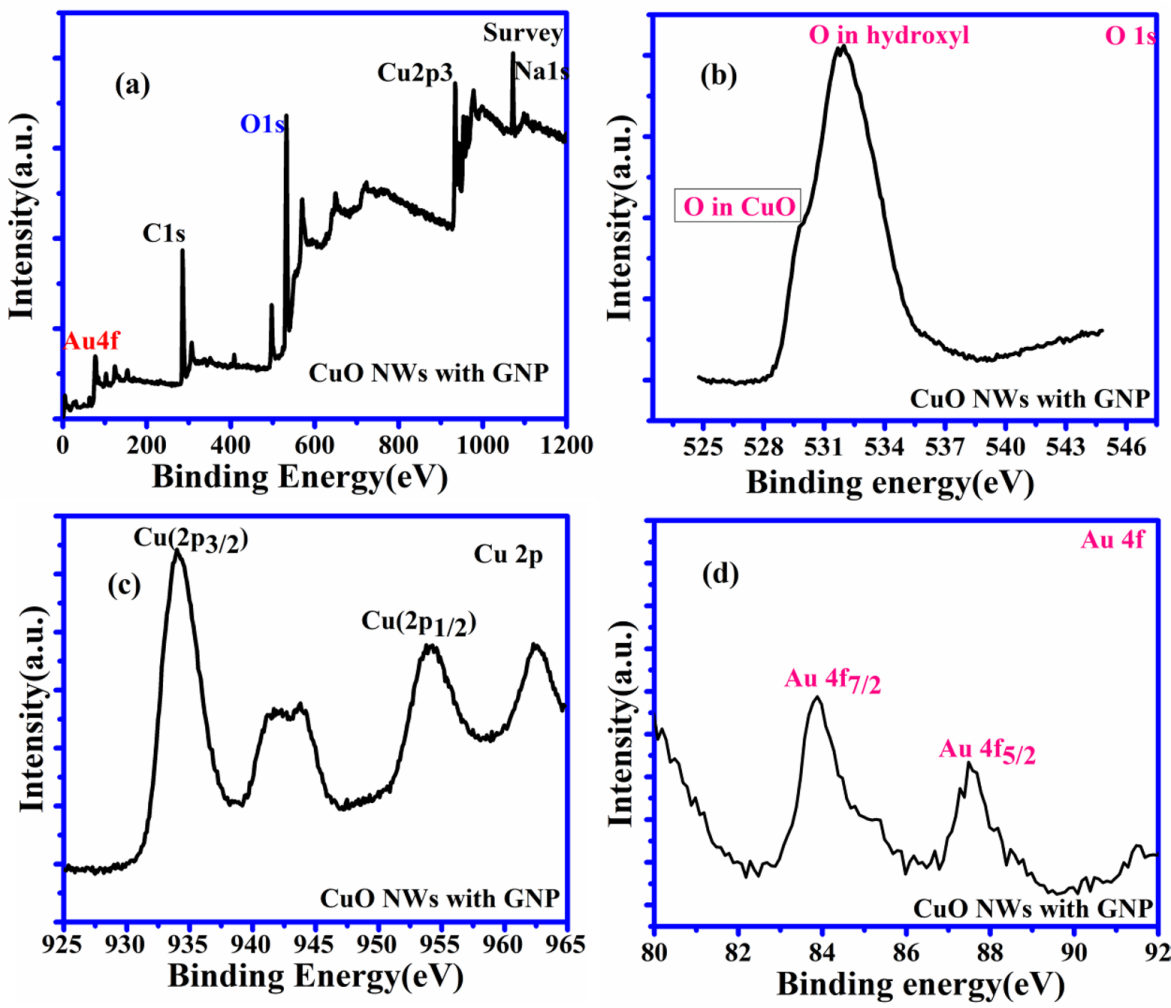
XPS analysis. (**a**) XPS spectrum of CuO NWs with GNP showing full scan survey (**b**) and corresponding de-convoluted peaks in the high-resolution spectra for O 1 s (**c**) Cu 2p (**d**) Au 4f.

The characterization details of the surface composition of CuO NWs and CuONWs with GNP (gold nanoparticles) were shown in Fig. 3. The whole spectrum of CuO NWs GNP shows the presence of Cu, O, Au, C, and Na atoms, which is shown in Fig. 3(a). The high resolutions spectra for O 1 s are shown in Fig. 3(b) which depicts two peaks in CuO NWs GNP at 531.16 eV and 532.27 eV equivalent to the oxygen vacancies or defect (O_V_)present because of O_2_ species in the lattice (O_L_) and dissociated (O_C_) oxygen species^40–42^. The XPS of Cu 2p core level is expressed in Fig. 3(c) where two peaks of energy 934 eV and 954 eV are shown in Fig. 3(c) which corresponds to Cu 2p_3/2_ and Cu 2p_1/2_ respectively which confirms the existence of the Cu^+2^. Two more peaks present at 941.8 eV and 961.9 eV are the satellite peaks of Cu2p_3/2_ and Cu2p_1/2_ respectively. They show the presence of the unfilled shell of 3d which again proves the presences of Cu^+2^ in the sample. Gold (Au) 4f core of the Au/CuO nanostructure (shown in Fig. 3(d) has been filled with two peaks i.e. Au 4f_7/2_ and Au 4f_5/2_ with binding energy 84.0 eV and 87.7eVwhich prove the presences of gold on CuO nanowires. For the selection of the better electrode the different copper oxide has been chosen like Cu (OH) _2_ NWs, CuO NWs and CuO NWs with GNP. The current which is observed in the case of Cu(OH)_2_ NWs is very low, moderate for CuO NWs and extremely high for CuO NWs with GNP in the solution of 0.5 M NaOH and 1 mM glucose concentrations.

**Figure 4:**
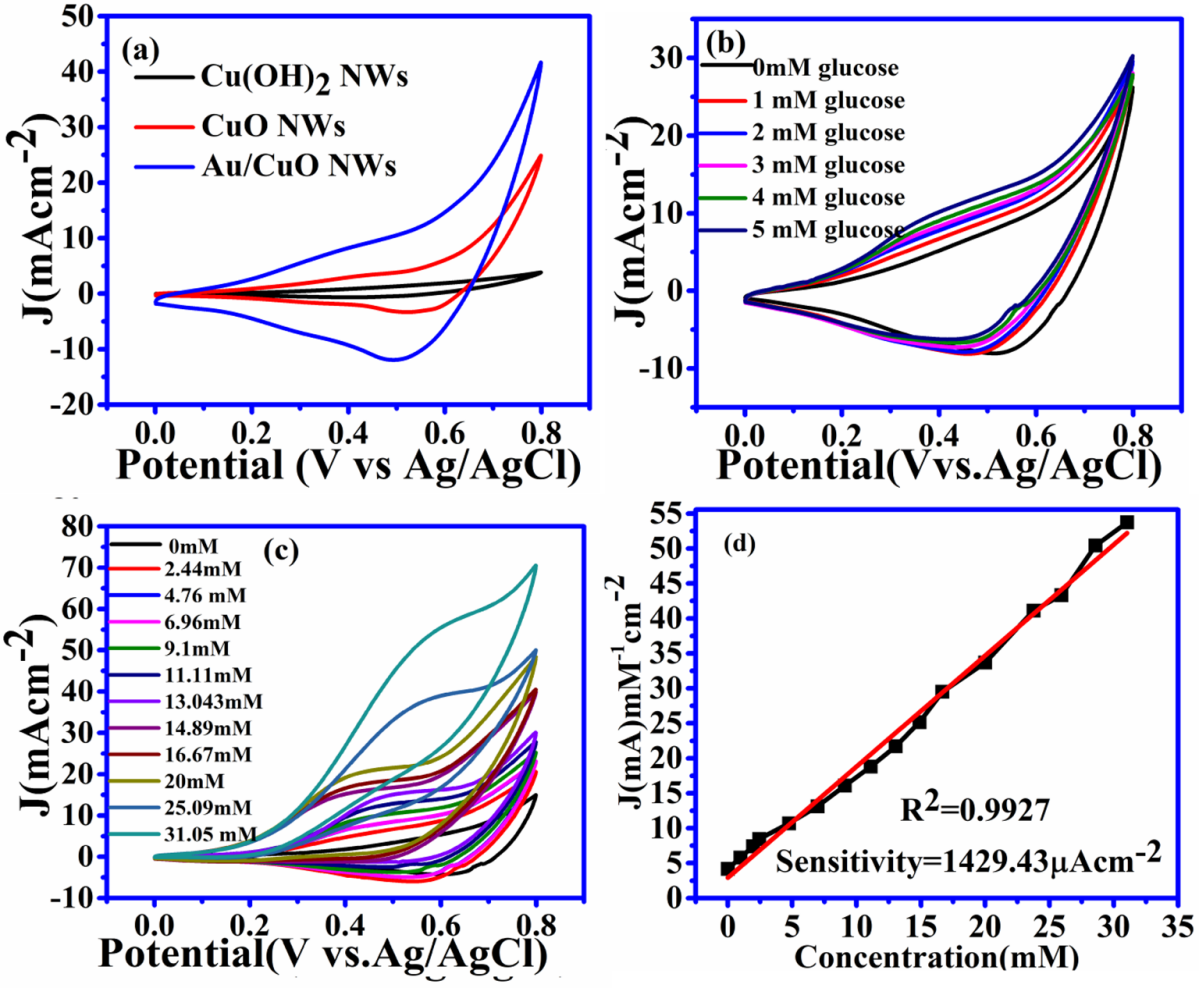
C–V curve of the electrode.(**a**) Current density versus potential graph for different type of electrode. (**b**) Current density of CuO NWs electrode with different concentration of glucose from 0 to 5 mM. (**c**) CV cure of Au/CuO type electrode with different concentration from 0 to 31.05 mM.(d)Corresponding calibration between concentration and current density at 0.55 V of reference voltages.

Due to the very high current observed in CuO NWs with GNP, we can use it as a working electrode for this research work. The drastic improvement with CuO NWs with GNP is due to intensive increments in surface to volume ratio for CuO NWs with GNP electrode. The C–V graph of the different working electrodes has been shown in Fig. 4(a).

CuO NWs with GNP electrode are dipped in the solution of 0.5 M NaOH with different concentrations of glucose at 0 mM, 1 mM, 2 mM, 3 mM 4 mM, and 5 mM. It is observed that the current increases with an increase in glucose concentration and linearity is found to be maintained as elucidated in Fig. 4(b). Figure 4(c) again shows the C–V plot same as Fig. 4(b) which covers the complete range of glucose for the working electrode at a solution concentration of 0.5 M NaOH up to till 31.05 mM which linearity is maintained. The final relation between current and concentration is shown in Fig. 4(d). It shows the sensitivity of the sensors 1429.43 µA cm^−2^ with R^2^ = 0.9927 and linearity shows a maximum value of 31.05 mM.

**Figure 5:**
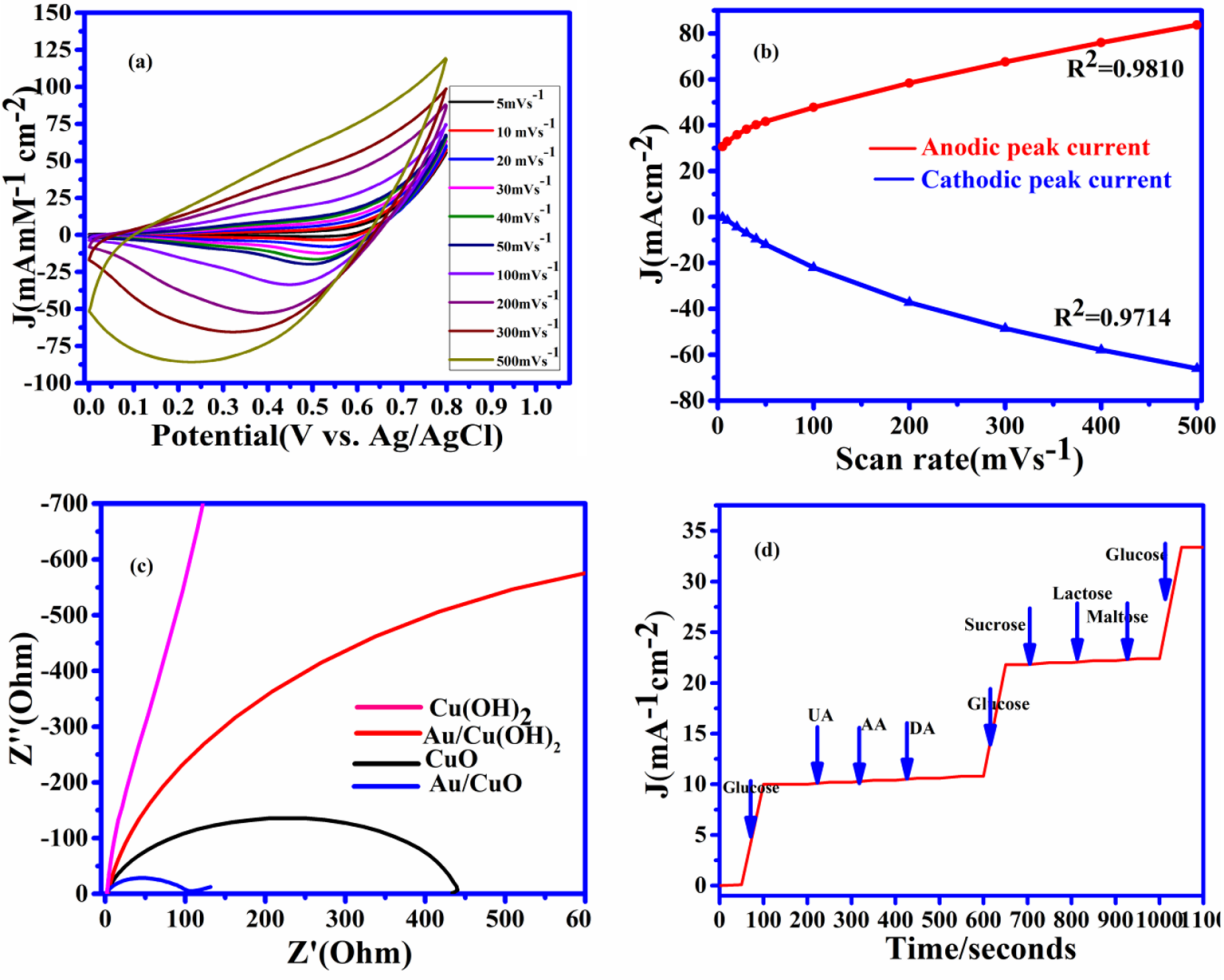
Electrochemical characterization 5 (**a**) C–V obtain at 1 mM glucose in 0.5 M NaOH solution at different scan rates. (**b**) The graph between peak current density versus scan rate (**c**) Nyquist plot for different copper electrode Cu(OH)_2_NWs, Au/CuO NWs, CuONWs, and Au/CuO NWs at OCP(open circuit potential). (**d**) Anti-interference property of the CuO NWs decorated by GNP electrode with 1 mM of glucose and with 0.1 mM each of UA, AA, and DA. Then 1 mM of glucose with 0.05 mM sucrose, lactose, and maltose and 1 mM of glucose added in the last.

The current–voltage graphs for the GNPs modified CuO NWs based electrodes with different scan rates are shown in Fig. 5(a). This figure confirms the redox reaction model. The linearity graph of the cathodic peak current and anodic peak current with different scan rates (5 mV s^−1^ to 500 mV s^−1^) is shown in Fig. 5(b). In view of the above discussion, it can be easily observed that the redox reaction model is surface-confined process^43,44^and the glucose molecule directly oxidizes the composite surface of CuO NWs with GNP and the electrons were directly transferred without any mediators.

The studied electronics properties over the surface are corroborated from the measurements of the electrochemical impedance spectra (EIS). EIS measurements are clearly shown in Fig. 5(c) depicts the gradual enhancement of conductivity from Cu (OH) _2_NWs electrode to CuO NWs with the GNP electrode. Figure 5(c) also reveals that the resistance is getting reduced and conductivity is getting enhanced due to decoration of gold nanoparticles on CuO NWs. EIS measurements have been taken place by considering a frequency range from frequency 10^5^ Hz –0.1 Hz with an open circuit potential of 26 mV. Nyquist plot of the (Cu (OH)_2_) NWs, (Au/Cu (OH) _2_) NWs, Copper Oxide (CuO ) NWs, and CuO NWs with GNP are shown in Fig. 5(c). The smallest semicircle of CuO NWs with GNPclearly shows the lowest resistance and higher conductance which confirms the accelerated rate of electrons transfer in the presence of gold nanoparticles.

Uric acid (UA), ascorbic acid (AA), dopamine (DA), sucrose, lactose, and maltose are the various interferences components along with glucose present in human blood serum. The concentration glucose in human blood serum is approximately 30 times more than the components of the above interference^45,46^. Due to the aforesaid reasons, the selectivity of CuO NWs with GNP electrode-based glucose sensor under study was examined at 1 mM glucose with these interfering elements of 0.2 mM each of lactose, sucrose, and maltose, UA, AA, and DA. Due to increased oxidation of glucose in comparison to other interferences components, the change in current was significantly high for glucose incomparison to other interferences species^47^which is as shown in Fig. 5(d). At the concentration of 1 M NaOH solution, working electrode CuO NWs with GNP has been used for measuring the current with an increase in glucose concentration. At the fixed voltage of 0.55 V, the increment in current was directly proportional to the amount of glucose added in the solution.

#### Reusability, reproducibility, and stability tests

The important factors for measuring the efficiency of the sensing devices are reusability, reproducibility, and stability. For the reusability, CuO NWs with the GNP electrode was dipped into a freshly prepared sample containing 1 mM of glucose in 0.5 M NaOH solutions at 50 mVs^−1^ scan rate and the resultant data were shown in Supplementary Fig. S3(a) and (b). The electrode was dipped in the solution for at least 10 times in the same solution or 10 different solutions. It is observed that CuO NWs GNP glucose sensing electrode retains more than 99% of its original response which shows its reusability. To test reproducibility, 10 freshly preparedAu/CuONWs with GNP electrode have been used in 1 mM of glucose solution of 0.5 M NaOH with a scan rate of 50 mV s^−1^. The peak current of each electrode is mention in the C–V graph as shown in Fig. Supplementary Fig. S3(c) and (d). The response of these electrodes is found to have a relative standard deviation of 5%. It can be concluded from the above discussion that the sensors based on CuO NWs with GNP electrode are good enough to reproduce approximately the same results which show the sensor reproducibility. To add more functionality to the sensor, stability test was evaluated by the C–V response of the CuONWs with the GNP electrode at an interval of 3 days for a month as shown in Supplementary Fig. S3 (e). The electrode was stored at room temperature upto the completion of the measurement. After the completion of 30 days, the response of the proposed CuO NWs with the GNP electrode was compared with the response of the electrode on the first day. The result is shown in Supplementary Fig. S3(f) as a histogram plot which conveys the information that the proposed sensor is very much stable which is able to retain 95% (measured on day 30) of its originals response (measured on day 0). The excellent stability response of the electrode was due to the stable grown of CuO NWs with GNP on the surface of the electrode which provides strong mechanical stability to the sensing device.

**Figure 6:**
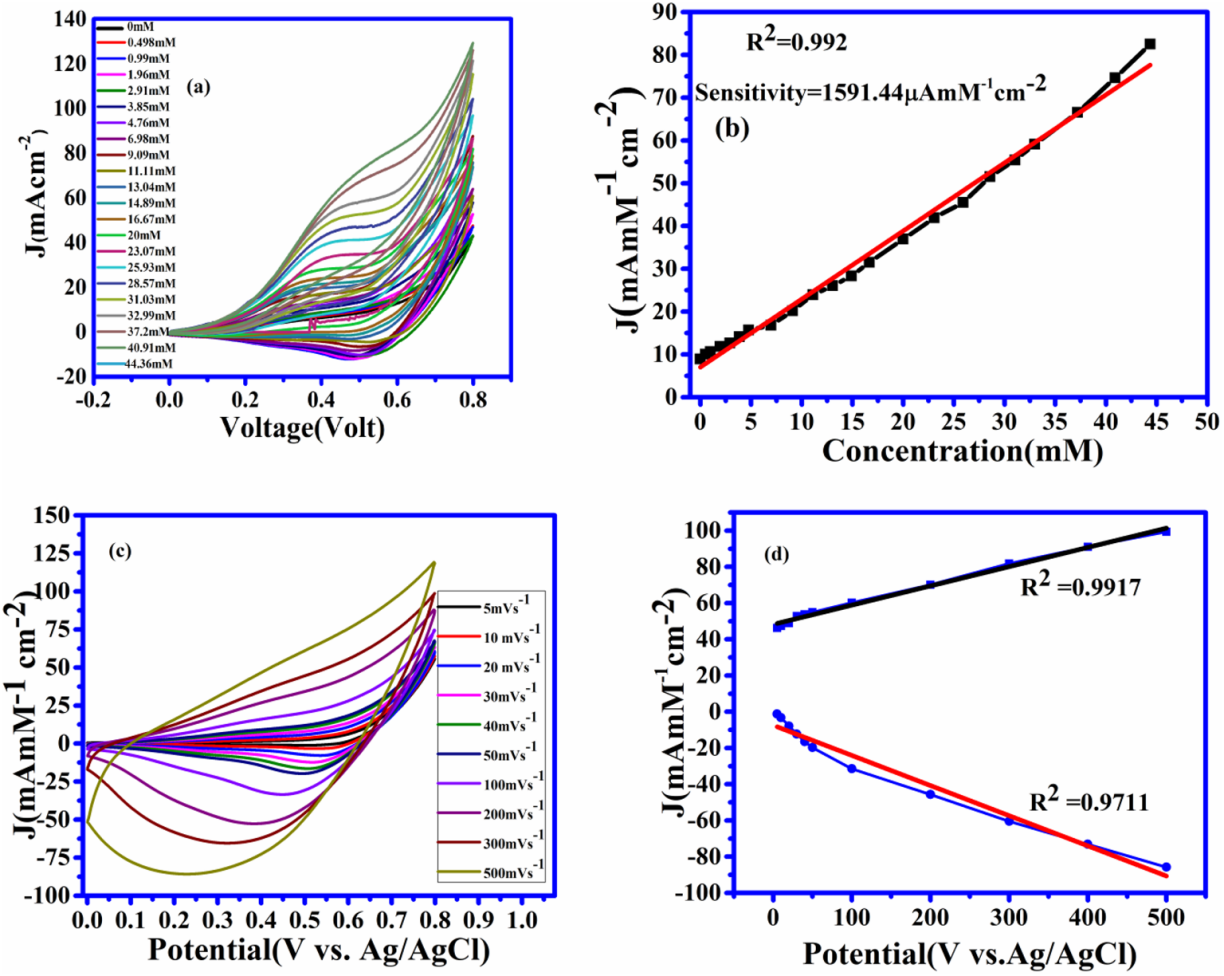
(**a**) C–V graph at 1 M NaOH solution with successive addition of glucose from 0 mM to 44.36 mM glucose solution. (**b**) Linearity graph between current density and concentration at 0.55 V of the reference voltage. (**c**) C–V graph with 1 M NaOH solution and 1 mM glucose concentration with different scan rates. (**d**) The linearity graph between current density and potential with different scan rates.

In all the above discussion the C–V measurement is done at 0.5 M NaOH. To test the sensor feasibility, we have also measured the C–V characteristics at 1 M NaOH solution as shown in Fig. 6(a) along with different concentrations of glucose. At a fixed potential of 0.55 V and the scan rate of 50 mV s^−1^, it is observed that the increment in current density is directly proportional to the amount of glucose added in the solutions. The linearity between current density and glucose concentration is shown in Fig. 6(b). The sensitivity of 1591.44 mA M^−1^ cm^-2^ with a wide linear range up to 44.36 mM is observed for the proposed sensor with 1 M NaOH. Due to this significant improvement in linearity with 1 M NaOH, the sensor is capable enough to detect the glucose level in highly diabetic patients. Figure 6(c) shows the C–V characteristics with different scan rates. The significance of this plot is to show that the proposed sensor is also showing linearity with varying scan rates. Figure 6(d) shows C–V characteristics with varying scan rates keeping the potential (voltage) constant. Here also we can observe that the linearity is still maintained by varying scan rates.

Sensitivity and linearity of the different Au-nanoparticles decorated CuO NWs (working electrode) have been given in Table 1. From the Table 1 it can be easily conclude that at 0.5 M and 1M NaOH solutions, this sensors are works for an extremely serious diabetic patients(linearity up to 31.05 mM and up to 44.36 mM) with good sensitivity.

To see the practical application of proposed device, real blood samples have been taken from our research group for testing in a private pathology laboratory named Dr. Lal Pathology Lab just located outside of our institute. The real time blood reports have been compared with the results obtained from our proposed sensor in Table 2. Approximately 100 μL blood is added into 9.90 ml of 0.5 M NaOH solutions, and the current density-concentrations responses are measured at Voltage = 0.55 V. The blood glucose concentration in the human blood serum is measured through the calibrated curve shown in Fig. 4(d). Each sample has been used 3 times and the average value has been taken for the comparison. It has been observed that our proposed sensor data are closely matched with the data provided by the Dr. Lal Pathology Lab. The maximum difference between our proposed sensor data and the data provided by the Lal Pathology Lab is ~ 3.6%. Thus, we safely conclude that our proposed sensor can be used for commercial glucose sensing applications.

**Table 1:** Comparasion of the non-enzymatic glucose sensors based on direct modification of working electrode.

Sample	Sensitivity (µA mM^−1^ cm^-2^)	Linear range (mM)	Detection limit (µM)	Solution concentrations (M)
CuO NWs with(GNP)^38^	4,398.8	0.005–5.9	0.5	0.1
CuO nanowires/ copper foam^27^	2,217.4 l	0.001–18.8	0.3	1
Au/CuO nanocauliflower^37^	708.7	0.001–30	0.3	1
3D Cu@ Cu_2_O aerogels^48^	194.88	0.001–17.12	0.6	0.1
Cuo Nano Particles/Ag^49^	2,762.5	0.05–18.45	0.5	0.0001
ZnO Nano rod decorated by CuONPs^50^	2,961.8	0.001–8.45	0.40	0.1
CuO NWs with Au NPs (This work)	1,425.69	0.001–31.05	0.3	0.5
CuO NWs with Au NPs (This work)	1591.44	0.001–44.36	0.3	1

In the summary, a facile step is involved in the fabrication of GNPs modified CuO NWs electrode-based non-enzymatic glucose sensors. GNPs coated CuO NWs enhance the effective surface-to-volume ratio of the electrode with respect only CuO, NWs based electrodes. This improves the catalytic property of the electrodes which, in turn, enhances the oxidation and reduction properties of the GNPs coated CuO NWs electrode. These enhanced properties in the proposed sensor gives a sensitivity of 1591.44 µA mM^−1^ cm^−2^ and 1,440.63 µA mM^−1^ cm^−2^ at a concentration of 1 M and 0.5 M NaOH, respectively. The linearity ranges of the glucose sensor are 44.36 mM and 31.05 mM at 1M and 0.5 M NaOH solvent concentrations respectively. The low detection limit of the sensor at different concentrations shows that the sensors can be used to detect extremely low glucose levels in salvia and urine. The results reported here are highly accurate and stable.

**Table 2:** Detections of glucose in blood samples.

Sample	Glucose concentration(mM) Spectrophotometric method(provided by health centre)	Proposed method(mM)	Recovery%	Error%
1	6.847(123 mg/dl)	6.6	96.4	3.6
2	5.34(97 mg/dl)	5.21	97.56	2.44
3	5.73(103 mg/dl)	5.64	98.42	1.57

## Experimental section

The fabrication and characterization methods of the electrode are nearly similar to our previous works reported elsewhere^38^. However, we have briefly discussed the fabrication and characterization of the electrode here for the clarity of the readers. We have also included some new characterization results for the better understanding of the readers.

## Materials

Alfa Aesar, Thermo Fisher Scientific (India) has provided the highly pure copper foil (99.9%) used as substrate for CuO NWs. Sodium hydroxide (NaOH), hydrochloric acid (HCl), ammonium persulfate [(NH_4_)_2_S_2_O_8_], acetone, isopropanol, and Malt extract powder have been bought from Merck Life Science Private Limited (India). Sisco Research Laboratories Private Limited (India) has provided glucose, sucrose, and uric acid. All the chemicals used are ultra-pure and of analytical grade, hence there is no need for further cleaning. DI water of high resistivity (18MΩ-cm) obtained from Merck Millipore system is used for cleaning purposes.

### Electrodes preparation

#### Formation of Cu (OH)_2_ NWs electrode

Small pieces of (5 mm × 5 mm) of copper foil have been prepared from the bulk copper sheet. These copper foils are first cleaned ultrasonically in DI water and HCl. Again these copper foils have been cleaned by acetone and isopropanol sequentially. After the completion of the cleaning process, these foils have been dried in the air. These foils are then dipped in a solution consisting of180 µl of DI water(H_2_O), 80 µl of 10 M Sodium Hydroxide(NaOH), and 40 µl of 1 M Ammonium Thiosulphate(NH_4_)_2_S_2_O_8_. After the completion of half an hour, these foils are taken out from the solution and dried in the flow of air. A deep blue film of Cu (OH) _2_NWs on Cu foils has been obtained^33^.

1 $${\text{Cu}} + 2{\text{NaOH}} + \left( {{\text{NH4}}} \right)_{2} {\text{S}}_{{\text{2}}} {\text{O}}_{8} \to {\text{Cu(OH)}}_{2} + {\text{Na}}_{2} {\text{SO}}_{4} + 2{\text{NH}}_{3} + 2{\text{H}}_{{\text{2}}} {\text{O}}$$

#### Formation of CuO NWs electrode

Deep blue colors of Cu(OH)_2_ on Cu foil were kept in alumina boat inside the furnace till half an hour in presences of Ar gas. The flow of Ar gas has been stopped after 30 min and foils are heated at 120 °C for three hours. For better crystallization, the foils were heated further at temapture180°C for two hours. The blue film of copper foils converted into black one and this is CuO NWs in the Cu foils ^33^.

2 $${\text{Cu}}\left( {{\text{OH}}} \right)_{2} \to {\text{CuO}} + {\text{H}}_{{\text{2}}} {\text{O}}$$

#### Formation of gold NPs decorated CuO NWs electrode

CuO NWs were dipped in the solution of 3 ml DI water containing 8 mg tetra chloro auric Acid (HAuCl_4_) for 10 min. After that rinse these foil in DI water. Again these foils were dipped in a solution containing 9 mg sodium borohydride (NaBH_4_) dissolve in 3 ml methanol. Finally, the foil is again rinsed with DI water for 2 min, to get gold nanoparticle decorated CuO NWs electrode on copper foil. The following reaction takes place during the formation of a gold nanoparticle on the CuO NWs electrode^39^.

3 $${\text{NaBH}}_{{\text{4}}} + 4{\text{CH}}_{3} {\text{OH}} \Leftrightarrow {\text{NaB}}\left( {{\text{OCH}}_{{\text{3}}} } \right)_{4} + 4{\text{H}}_{2}$$

4 $${\text{4HAuCl}}_{{\text{4}}} + 3{\text{NaBH}}_{{\text{4}}} \to 4{\text{Au}} + {\text{6H}}_{{\text{2}}} + {\text{3NaCl}} + 3{\text{BCl}}_{3} + 4{\text{HCl}}$$

#### Electrode characterizations and electrochemical set-up

The copper foils, Cu(OH)_2_NWs, CuO NWs, and GNP modified CuO NWs are characterized using X-ray diffraction (XRD) (RIGAKU-SmartXDMAX, PC-20, 18-kW Cu rotating anode, Rigaku, Tokyo). The surface morphology of GNP modified CuO NWs is investigated by FE-SEM (Model-Nova Nano SEM FEI Company of USA (S.E.A.) PTE, LTD) and (HR-TEM) (Model-Tecnai G2 20 TWIN FEI Company of USA (S.E.A.) PTE, LTD).The electrochemical measurements were performed in a 3-electrode electrochemical cell configuration with CuO based electrodes as working electrodes, a platinum wire as counter electrode, and Ag/AgCl as a reference electrode with an electrochemical workstation (ModelCS: 350, S/N: 1,609,178, Corr test Instrument, China). The electrode preparation processes have been shown in Fig. 1(a) in which the steps involved in the formation of CuO NWs with GNP have been shown. Different concentration of NaOH (0.5 M and 1 M NaOH) solution has been used an electrolyte for measurement of cyclic voltammetry (C–V) at room temperature. In Fig. 1(b), bendable clean strip of copper foil is shown which is used in Fig. 1(a). 

## Supplementary information

Supplementary information
