# Author Correction: Au nanoparticles modified CuO nanowire electrode based non-enzymatic glucose detection with improved linearity

**DOI:** 10.1038/s41598-020-74222-6

**Published:** 2020-10-14

**Authors:** Ashwini Kumar Mishra, Deepak Kumar Jarwal, Bratindranath Mukherjee, Amit Kumar, Smrity Ratan, Manas Ranjan Tripathy, Satyabrata Jit

**Affiliations:** 1 Department of Electronics Engineering, Indian Institute of Technology (BHU) Varanasi, Varanasi, 221005 India; 2 Department of Metallurgical Engineering, Indian Institute of Technology (BHU) Varanasi, Varanasi, India

Correction to: *Scientific Reports* 10.1038/s41598-020-67986-4, published online on 10 July 2020

The original version of this Article contained an error in the title of the paper, where the words “nanowire electrode” were incorrectly given as “nanowireelectrode”.

In addition, the original version of this Article contained typographical errors. As a result, in the Introduction,

“Electrochemical based glucose sensors are of two types depending on the electro-catalyst used on the sensor Electrodes^10^. The first is enzymatic type glucose sensors that requires an enzyme like glucose oxidase (Gox), GDH, etc. on the electrode while the second type is called non-enzymatic glucose sensors which requires no enzymes in electrode for detection^10,17,18^.”

now reads:

“Electrochemical glucose sensors are of two types depending on the electro-catalyst used on the sensor Electrodes^10^. The first is enzymatic type glucose sensors that requires an enzyme like glucose oxidase (GOx), GDH, etc. on the electrode while the second type is called non-enzymatic glucose sensors which requires no enzymes in electrode for detection^10,17,18^.”

“Further, the enzymatic glucose sensors are costlier than the non-enzymatic glucose sensors due to non-requirement of costly enzymes^20–23^.”

now reads:

“Further, the enzymatic glucose sensors are costlier than the non-enzymatic glucose sensors due to requirement of costly enzymes^20–23^.”

“In this article, we have demonstrated the improvement of linearity and sensitivity of the GNPs modified CuO NWs electrode based glucose sensor using the higher levels of solvent (NaOH) concentrations at 0.5 M and 1 M.”

now reads:

“In this article, we have demonstrated the improvement of linearity and sensitivity of the GNPs modified CuO NWs electrode based glucose sensor using the higher levels of electrolyte (NaOH) concentrations at 0.5 M and 1 M.”

In the Results and discussions section under subheading ‘Characterizations of electrodes’,

“The Field Emission Scanning electron microscopy (FE-SEM) images morphology of CuO NWs with GNP under study is shown in Fig. 1b with low resolution.”

now reads:

“The Field Emission Scanning electron microscopy (FE-SEM) images to illustrate morphology of CuO NWs with GNP under study are shown in Fig. 1b with low resolution.”

“Sead pattern shown in Supplementary Fig. S2(d) also confirms the absences of gold in the CuO NWs.”

now reads:

“SAED pattern shown in Supplementary Fig. S2(d) also confirms the absences of gold in the CuO NWs.”

“Two more peaks present at 941.8 eV and 961.9 eV are the satellite peaks of Cu2p3/2 and Cu2p1/2 respectively shows the presence of unfilled shell od 3d9 and it again proves the presences of Cu + 2 in the sample.”

now reads:

“Two more peaks present at 941.8 eV and 961.9 eV are the satellite peaks of Cu2p3/2 and Cu2p1/2 respectively. They show the presence of the unfilled shell of 3d which again proves the presence of Cu + 2 in the sample.”

“The studied electronics properties over the surface are a very important tool for the measurements of the electrochemical impedance spectra (EIS).”

now reads:

“The studied electronics properties over the surface are corroborated from the measurements of the electrochemical impedance spectra (EIS).”

In the Materials section under subheading ‘Electrodes preparation’,

“Finally, the foil is again revised with DI water for 2 min, to get gold nanoparticle decorated CuO NWs electrode on copper foil.”

now reads:

“Finally, the foil is again rinsed with DI water for 2 min, to get gold nanoparticle decorated CuO NWs electrode on copper foil.”

These errors have now been corrected in the PDF and HTML versions of the Article.

