# Supplementary material for: Au nanoparticles modified CuO nanowire electrode based non-enzymatic glucose detection with improved linearity
Source: Sci Rep. 2020 Jul 10;10:11451. doi: 10.1038/s41598-020-67986-4 (PMC7351779; doi:10.1038/s41598-020-67986-4)
Supplement: Supplementary file 1 — Supplementary information [file 41598_2020_67986_MOESM1_ESM.docx]

**Au nanoparticles modified CuO Nanowire electrode based non-enzymatic glucose detection with improved linearity**

**Ashwini Kumar Mishra^1^, Deepak Kumar Jarwal^1^, Bratindranath Mukherjee^2^, Amit Kumar^1^, Smrity Ratan^1^, ManasRanjanTriphaty, and Satyabrata Jit^1^***

1. The authors are with the Department of Electronics Engineering, Indian Institute of Technology (BHU) Varanasi Varanasi221005, India (*email:ashwanikm.rs.ece15@itbhu.ac.in;sjit.ece@itbhu.ac.in). 2. The author is with the Department of Metallurgical Engineering IIT (BHU) Varanasi.

**Corresponding Email- sjit.ece@iiitbhu.ac.in**

We shave shown the bright-field HRTEM images and corresponding SAED patterns of the Au modified CuO NWs and TEM image of CuO NWs as Figure S1 and Figure S2, respectively, in the supplementary file. It is clearly observed that spheroidal Au nanoparticles on CuO nanowires are clearly distinguished from mass-thickness contrast in bright field TEM images HR-TEM image of the heterostructure. On the other hand, the selected area electron diffraction (SAED) pattern shown in Figure S2(d) represents clearly only the CuO phase with no signature of Au phase.


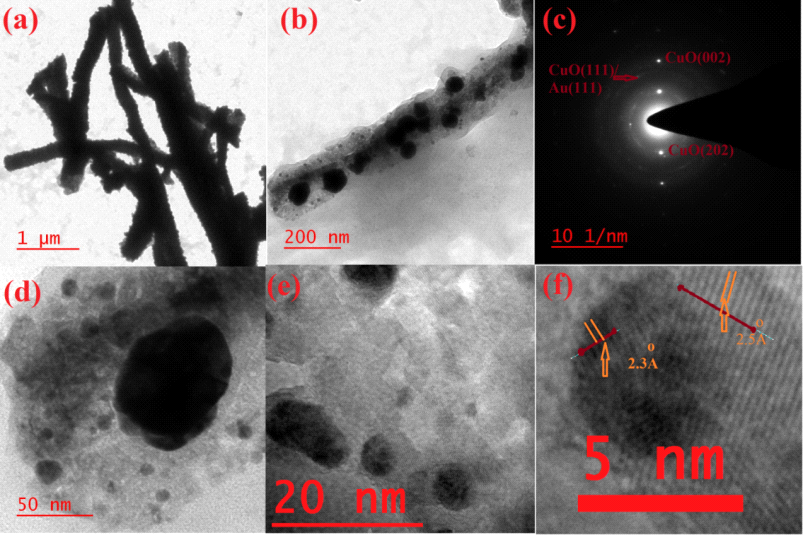


Figure S1: (a) The HRTEM images of freestanding Au modified CuO NWs, (b) magnified version of a single CuO NWs with gold nanoparticles, (c) SEAD pattern of selected area, (d) Moderate-resolution of Gold modified CuO NWs, (e) high resolution of a single NWs with GNP, (f) HRTEM of further magnified version of a single CuO NWs with GNP for d- spacing evolution.


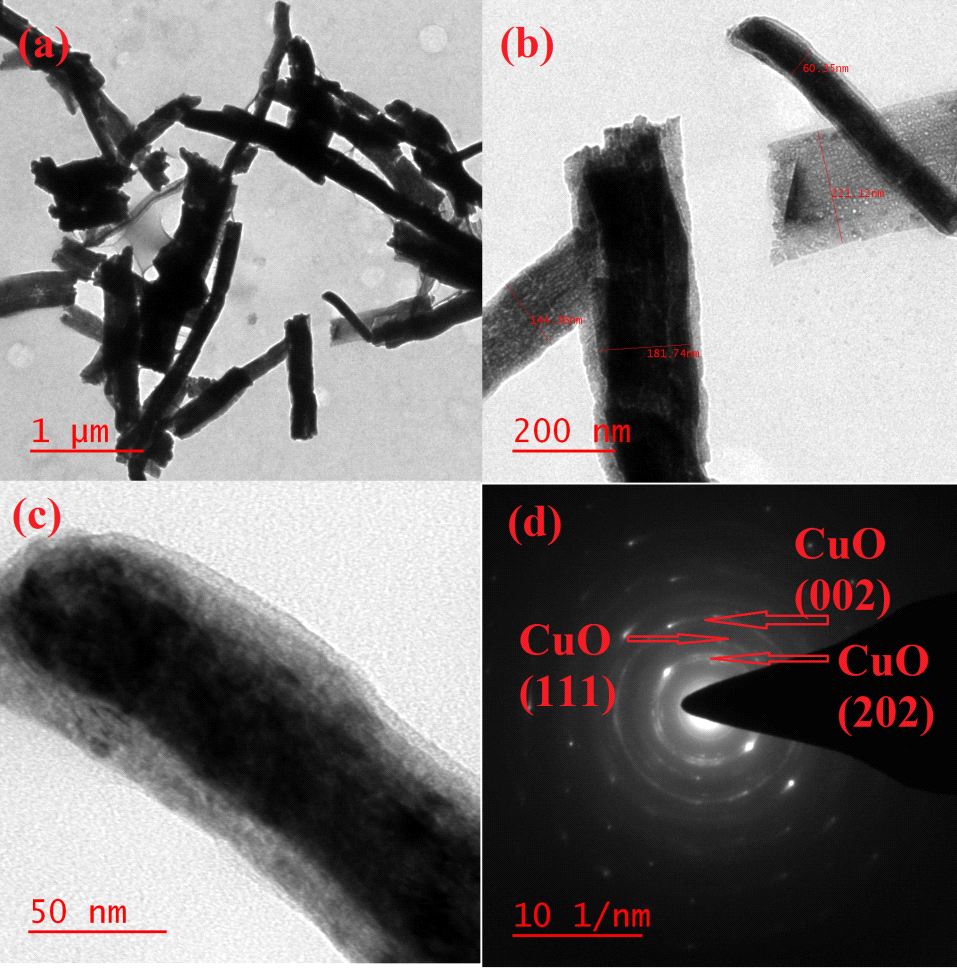
 Figure S2: Bright field TEM images at different magnifications are shown in (a-c). It can be observed from the images that CuO nanowires are smooth and no mass thickness contrast and corrugated morphology due to Au NPs as observed in Figure S1 are present. (d) Selected area electron diffraction (SAED) can be clearly indexed with CuO phase with no signature of Au phase.

Reproducibility analysis discussed in the manuscript are shown in Figure S3.


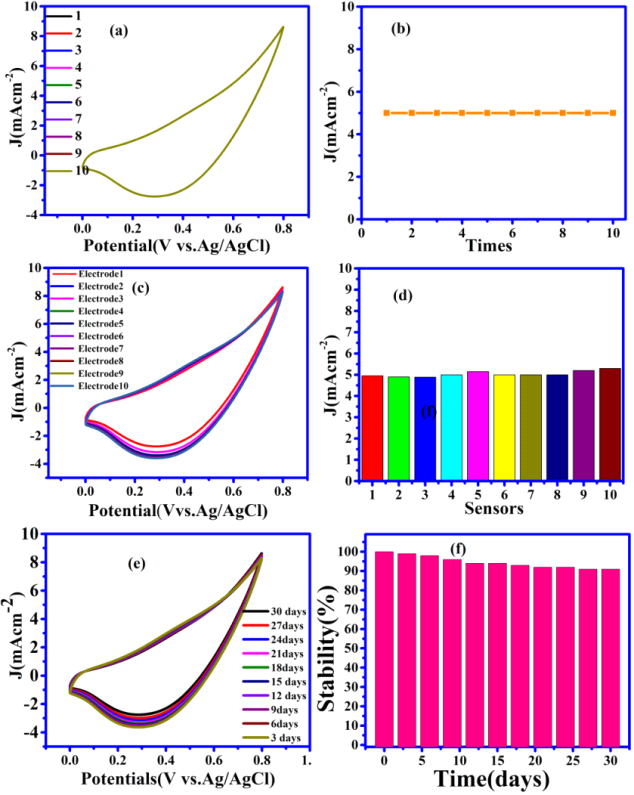


**Figures S3:(a)** Reusability graph between at 0.5M NaOH and 1mM glucose concentration (b) constant current of 5mA obtained at 0.55vollt potential(c)Reproducibility graph from 1 electrode to 10 electrode after 1mM addition of glucose(d) The histogram curve at 0.55V potential and 1mM glucose (e) C-V graph between taken between the interval of 3days and up to 30 days (f) corresponding histogram from 0 day up to 30 days with interval of 3 days.

The SEM-EDS area scan data have been shown in Figure S4. Note that ~20 atomic % of Au is observed in the EDS spectra.

| Element | Weight% | Atomic weight% |
| --- | --- | --- |
| Cu | 42.35 | 53.39 |
| O | 5.03 | 25.20 |
| Au | 52.62 | 21.40 |


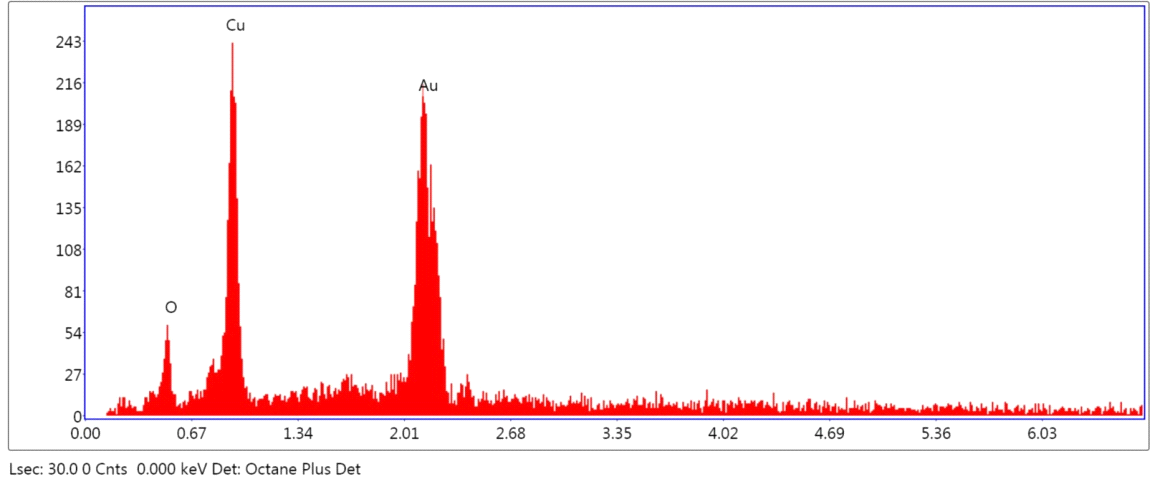


Figure S4: Composition of materials and EDS spectrum of CuONWs with gold nano particle shown in selected area
